# “No, but where are you *really* from?” Experiences of perceived discrimination and identity development among Asian Indian adolescents

**DOI:** 10.3389/fpubh.2022.955011

**Published:** 2022-10-18

**Authors:** Asha K. Unni, Jamilia J. Blake, Phia S. Salter, Wen Luo, Jeffrey Liew

**Affiliations:** ^1^Department of Educational Psychology, Texas A&M University, College Station, TX, United States; ^2^Department of Health Behavior, Center for Health Equity and Evaluation Research, Texas A&M University, College Station, TX, United States; ^3^Psychology, Africana Studies, Davidson College, Davidson, NC, United States

**Keywords:** South Asian, Asian Indian, race, ethnicity, discrimination, identity development

## Abstract

Asian Indians were the first South Asians to immigrate to the United States in the late 1800s and are currently the largest ethnic group of South Asians living in the United States. Despite this the literature on perceived ethnic and racial discrimination experiences among this group is relatively understudied. The documented experiences of Asian Indians who either recently immigrated from India or were born and raised in America pose an important question: what are the experiences of perceived discrimination among Asian Indians living in America, particularly among younger populations who are continuing to develop their racial and ethnic identities? The current study utilized phenomenological methodology to explore the experiences of nine Asian Indian American adolescents' (ages 12–17 years). Data were collected *via* semi-structured interviews to assess participants' experiences of ethnic and racial discrimination and identity development. Thematic analysis was used to identify themes and subthemes among the participants' responses. Asian Indian adolescents living in the United States report experiencing discrimination at a young age. It is also evident that Asian Indian youth experience significant challenges when developing their sense of ethnic and racial identity while living within the United States. Findings document the racial and ethnic discrimination that Asian Indian adolescents living in the United States may experience from a young age. Importantly, these discrimination experiences are occurring as Asian Indian adolescents are developing their racial and ethnic identities. This study provides insight for future research, which is necessary to fully understand the experiences of Asian Indian adolescents.

## Introduction

According to the U.S. Census, there are currently over 3.5 million South Asians living within the United States, occupying the fastest growing population among all major ethnic groups in the country (1). The growth of the South Asian population is partially due to the increasing number that are migrating to the U.S. for various educational and employment opportunities (2). Despite recent increases in immigration, South Asians have experienced significant institutional hurdles in their historical movement to the United States (3). The Barred Zone Act of 1917 and Asian Exclusion Act of 1924 significantly impacted immigration of South Asians to the United States, and although they were eventually allowed entry by quota in 1946, South Asians were still viewed as outsiders (3). The increased presence of South Asians ultimately led to tension amidst White communities in areas of high immigration, resulting in ethnic discrimination, which is the act of treating other individuals differently based on cultural values, beliefs, and practices as opposed to just their race alone (2, 4).

These tensions increased over the years, especially in the aftermath of the terrorist attacks on September 11th, 2001. Racial and religious markers (e.g., skin color and headscarves, respectively) were (mis)labeled as physical indicators of “terrorists”, resulting in racial discrimination directed at South Asians, which is differential treatment of individuals based on their qualities (such as skin color) associated with a particular racial group (2, 5). According to the FBI (6), trends in anti-Muslim hate crimes have risen by 67% from 2014 to 2015 and are currently at the highest level since 9/11 (7). It is estimated that these trends will continue to rise in the current political climate, where “xenophobic political rhetoric,” defined as “comments motivated by a fear or hatred of those perceived to be different, other, or ‘foreign',” become the norm (7) (p. 5). Given the alarmingly high rates of hate crimes directed at individuals perceived as foreign, it is essential to understand how South Asians living in America, or South Asian Americans (SAA), are currently experiencing and coping with discrimination.

Despite these rates of discrimination, the literature on SAAs' perceived experiences is relatively understudied (8). It is speculated that this may be due in part to the ‘model-minority myth,' attributed to most Asian American groups (9). The model-minority myth is based on stereotypes of Asian Americans as successful, high achieving, and well-off in society (9–11). The proportion of Asian Americans with less than a high school education, who are living in poverty, working overtime, have multiple jobs, or who experience income inequality, are overlooked in these cases of success, however (11, 12). A significant number of Asian Americans have less than a ninth-grade education; even those who are at the same educational level as White Americans are paid comparably less (11). Additionally, relatively recent studies have provided contrasting data that suggest Asian Americans do not differ significantly from other groups in terms of GPA, SAT scores, and selection of science and engineering majors (10). Yet, Asian Americans and members of other groups still internalize this stereotype considering the continued stereotypical portrayal of Asian Americans in mass media (10). This stereotype can lead to the misperception of Asian Americans as mostly immune to the racial discrimination other racial minority groups face, when in fact the data suggest that Asian American adults experience racial discrimination at rates comparable to Latinx and Native American individuals (13–15). Further, the few discrimination studies available focus on SAAs broadly, even though the South Asian label is pan-ethnic and includes various ethnicities, cultures, and religions. Asian Indians are currently the largest ethnic group of SAA living in the United States (1, 16). Thus, it is important to understand Asian Indians Americans' (AIA) unique experiences with discrimination in the broader context of South Asian experiences.

Kaduvettoor-Davidson and Inman (8) (p. 157) define first-generation SAA (which include AIAs) as “those who immigrated to the United States as adults, whereas second-generation South Asians Americans (and Asian American Indians) are those who are either born in the United States or immigrated prior to age 18.” India was colonized and under British imperial rule for 400 years, and this resulted in the internalization of cultural norms and values of the British, including the primary use of the English language and the perception of lighter skin being associated with higher social class (17). Although immigrating to the U.S. meant validating many of these same practices, it is not clear whether this made it easier or more difficult for Asian Indians to assimilate into American culture. Even with assimilation, AIAs are still being discriminated against racially and ethnically and are seen as perpetual foreigners. The historical context of AIAs' experiences with colonization suggests that first-generation immigrants are potentially experiencing both racial and ethnic discrimination.

While first-generation immigrants may experience discrimination due to observable cultural and ethnic factors such as clothing or accents, as well as race, it is possible that second-generation immigrants are mostly subjected to race-based discrimination (8). Second-generation immigrants within the United States are raised within a society that emphasizes race, a factor that has social, cultural, and political implications and expectations such as institutional racism, educational achievement, and employment disparities. This results in the racial socialization of second-generation immigrants based on their skin color, in addition to their ethnic identification (18). While most of the literature has discussed the discrimination experiences of first- and second-generation Asian Indians in a comparative sense, additional research is needed to fully understand how second-generation Asian Indians' discrimination experiences may be unique in nature.

Most of the studies conducted on AIAs and their perceived experiences of discrimination have focused on young adult and adult populations, neglecting investigation into adolescents' discrimination experiences. Tummala-Narra and colleagues (19) examined South Asian adolescents' narratives to understand acculturative stress and coping and found that several participants reported experiences of discrimination toward their family and themselves across multiple situations and contexts. Participants also reported facing stereotypes focused on terrorism and being model minorities, as well as feeling a general lack of belonging and acceptance among peers and outside the home. Similarly, South Asian adolescents in another study reported peers, teachers, and adults held higher academic expectations of them compared to other adolescents their age, and they cited this as a form of ethnic discrimination (20). The adolescents in this study also reported significantly higher peer-related distress, compared to their African American, Hispanic, and non-Hispanic White peers. Other studies indicate South Asian individuals experiencing heightened anxiety related to the environment, awareness of their physical appearance, alienation, emotional stress, issues with self-esteem, and depression (8, 21, 22).

Given SAAs' significant history of colonization and immigration to the U.S., Ibrahim and colleagues (3) developed a model to understand first-generation SAA identity in the context of specific cultural factors. Ibrahim et al. (3) asserted that first-generation SAA develop their identities within a larger social-cultural context that considers ethnic group of origin, community, religion, neighborhood, social class, educational level, gender, and sexual orientation. This factor of ethnic group of origin is what mainly distinguishes the South Asian Identity Development Model from other ethnic minority models currently available. Importantly, Ibrahim et al.'s (3) framework acknowledges the historical significance of the colonization, immigration, and discrimination experiences of first-generation SAAs as context for how individuals develop their sense of identity. Considering interethnic differences among SAAs, it may be difficult to generalize identity development among AIAs. However, Iwamoto and colleagues (23) assessed the application of Ibrahim et al.'s (3) framework among second-generation AIAs and found support for the idea of identity development being a continuous process across the lifespan, subject to individual contextual factors (i.e., racism, etc.). Identity is an important factor to consider for discrimination experiences because there are a number of studies that show ethnic and racial identities may play a protective role when it comes to the negative effects of discrimination (24).

The purpose of the current study is to expand on the current literature on the perceived racial and ethnic discrimination that second-generation AIA youth experience. It is clear that the experiences of AIA adolescents are largely understudied, since most of the studies included have focused on adult populations. Age appears to change outcomes of discrimination and the degree to which ethnic identity can serve as a protective factor. With the additional consideration that adolescence is a crucial time for identity development, there is a clear need to examine this population to understand how youth experience racial and ethnic discrimination. Clarification of the conceptualization and role of racial and ethnic identity for AIAs is warranted, given the mixed results among the discrimination literature. The definition of ethnic discrimination is often used interchangeably with racial discrimination, which prevents researchers from fully understanding AIAs' experiences. While many studies examine first-generation AIAs, the contextual factors that differentiate experiences of first- vs. second-generation AIAs also necessitates an exploration of the role of ethnic and racial identity among second-generation individuals.

## Materials and methods

### Research design

The lead researcher identifies as a second-generation AIA woman, and the research team is composed of scholars of color of African and East Asian descent. The lead researcher acknowledges her own negative experiences of discrimination based on her race and ethnicity. The lead researcher is interested in how these experiences are unique to younger individuals as they are beginning to form their identities in adolescence. The lead researcher wants to explore the lived experiences of second-generation AIA adolescents and how discrimination may impact these individuals' sense of mental well-being. Given the aforementioned gaps in the literature and the authors' positionalities, the current study will utilize a phenomenological methodology, with an interpretivist approach. Phenomenological methodology allows the researcher to understand a given phenomenon, in this case, discrimination, as lived experiences that are unique to each individual (24). An interpretivist approach is one where “the researcher is trying to make sense of the participants trying to make sense of their world” through their own interpretations (25).

The current study has three main questions. (1) “What are second-generation Asian Indian American youth's experiences with racial and ethnic discrimination;” (2) “How are these unique discrimination experiences impacting these individuals;” and (3) “What are second-generation Asian Indian American youth's experiences with forming their racial and ethnic identities?” These questions are exploratory in nature and will provide information on the nature of discrimination and how identity formation is experienced by second-generation Asian Indian American adolescents.

### Sample and recruitment

The present sample consisted of nine adolescents ages 12–17 years recruited from the Southwestern region of the United States. These participants were selected based on their identification as second-generation AIAs. For the purpose of this study, second-generation AIAs are defined as individuals who were born in the United States, to parents who immigrated from India after the age of 18. Snowball recruiting methods, internet posts, and recruitment flyers shared through local leaders at churches, temples, mosques, and cultural centers in major metropolitan cities in the Southwest region of the U.S. Parental consent and child assent were obtained prior to the interviews, consistent with institutional Internal Review Board (IRB) approval.

### Measures

Data were collected *via* an individual 30 min to 1 h semi-structured interview with participants. The interview included questions about participants' self-defined sense of ethnic and racial identity, their cultural background and history of acculturation in the context of their families, the extent of their interactions with AIA and non-AIA peers, and their experiences with various forms of discrimination. Five hypothetical scenarios related to discrimination to understand how each participant uniquely defines and frames discrimination from their own perspectives was provided along with follow-up questions and prompts as necessary. Interviews were conducted in person, or *via* Doxy.me, a HIPAA-compliant videoconferencing service. Interviews were audio recorded and transcribed through a third-party service (Rev.com) to support later coding and analysis.

### Study procedures

Interested parents set up an initial meeting in person or virtually *via* Doxy.me to review study materials, to have questions or concerns addressed, and to complete consent forms. Once parent and participant consent and assent were obtained, interviews with the adolescents were scheduled through parents. Each participant was compensated with a $20 Amazon giftcard after completion of the interviews.

### Analysis

The current study employed thematic analysis using NVivo software (26). Thematic analysis allowed for the participants to provide full accounts of their experiences with discrimination, to identify themes that emerged across participants' data. Rev.com (third-party transcription service) transcribed each participant's interview, and transcripts were sent to participants for member checking, allowing adolescents to review contents for validity (27). Content of the interviews were then reduced to examine surface themes and paraphrases, and themes were further explored for subthemes, allowing us to map out how themes and subthemes were interrelated. We then identified how themes were common across participants' interviews to explain how second-generation AIAs adolescents are experiencing discrimination.

## Results

For the current study, nine Asian Indian American adolescents were interviewed; demographics of the participants are listed in Table 1. NVivo software was used for coding (26).

**Table 1:** Demographic characteristics of interview participants.

**Participant Initials**	**Age**	**Grade**	**Gender**	**Generation Status**
A.T.	16	11th	M	2^nd^
L.G.	15	10th	F	2^nd^
S.C.	13	8th	M	2^nd^
N.D.	14	8th	F	1.5^a^
M.K.	12	7th	F	2^nd^
R.V.	15	10th	M	2^nd^
H.G.	14	9th	F	2^nd^
N.V.	15	9th	F	2^nd^
S.M.	17	12th	F	2^nd^

^a^1.5 generation Asian Indian adolescents are those who were born in India but came to the U.S. before age 16 (9). This participant moved to the U.S. at age 4.

There were three major themes that emerged across the nine interviews out of the prompted scenarios: discrimination, aspects of racial and ethnic identity, and the balancing act of identity management. There were also several subthemes identified; with “Otherness,” “Racial discrimination,” and “Assimilation” appearing as the top three subthemes. These themes and subthemes arose both in response to the scenario prompts and to the questions asked about participant experiences.

### Discrimination

Within the discrimination theme, several subthemes appeared during coding. In particular, “racial discrimination,” “ethnic discrimination,” “in-group discrimination,” “microaggressions,” “terrorist,” “Trump,” and “fear of discrimination” were all subthemes identified across most of the interviews. Some of the participants recalled specific scenarios that stirred up feelings of fears of discrimination such as recent hate crimes against Indians and increased activity of white supremacist groups. One participant commented on how he self-identifies, “…like in Kansas, an Indian man was shot…in a bar. After that I got really scared because like, I'm brown, I'm Indian, living in America and I didn't want anything to happen tragic to me or my family. So before saying I was Indian, I would always say I'm American and also after the…election…I would always just identify as American after that.” Similarly, another participant commented on hurtful comments she would hear, “After…was elected in 6th grade, everyone was really glad ‘cause they thought that he would build a wall to kick out all the Mexican people and the Indian people…they were the people that bullied my *hijabi* friend. They talk a lot about Indians and how they're part of ISIS or something.”

With ethnic discrimination, participants cited experiences of peers misunderstanding and making assumptions based on language, religion, and culture. “…this one kid…found a rock and said ‘look it's your God”' and “…in math class…we had like little dots and we would have to...put them into groups... And a white kid was saying ‘is this your God?' and put it on his forehead.” Other hurtful comments often came up among peer interactions—“…and then sometimes they would say stuff about the food or they would mock an Indian accent…like, ‘I don't like Indian food'… some people have said things like ‘it's gross' or ‘it's weird' or ‘it smells really bad.”' Some of these interactions can be identified more accurately as microaggressions, as well. As one participant experienced related to academics, “…you don't do as well as you think you would on a test or something and someone else would be like… ‘How? You're Indian, you're supposed to be doing good…you're supposed to have an A.'…and that kind of hurts.” Comments like these are consistent with endorsement of the model minority myth.

The issue of skin color was another form of discrimination expressed by some participants, mostly among AIA peers. “[Indians] think that light-skinned Indians are more superior to dark-skinned Indians. They always want me to keep my light skin or whatever.” Another participant said “…I love my skin color...Indian society...tells you that only fair, skinny girls are pretty…being darker-skinned colored, I have been called the color of dirt before, and by a fellow Indian…and it really did hurt me.” Interestingly, the references made to skin color were only brought up among the female participants and were often referencing themes of beauty and desirability. Although participants did not consistently identify experiences as discriminatory or racist, they were often described as feeling hurtful.

### Racial and ethnic identity

Most of the participants used race and ethnicity as interchangeable concepts. Many participants mentioned that they were first aware of their racial and/or ethnic identity early on in their school years. Often these experiences involved comparisons of skin color, or interactions where peers questioned aspects of the participants' culture. Participants reported feeling embarrassed about participating in Indian classical dance, having religious calendars inside their home, or deciding to spend more time speaking English instead of their mother tongue. Parents' strict parenting styles (e.g., participants were not allowed to attend sleepovers or go to pool parties) were also experiences that made participants aware of their differences among their friends. Some participants reported feeling angry that they did not have white skin like their friends, and their desire to be more “American” instead as early as preschool.

Several participants spoke about having AIA friends and how comfort changed across the years for some individuals. Most agreed that having Indian friends was more comforting because it was easier to relate and be accepting of others' values and beliefs. In fact, a participant said her mother insisted that she continues to keep Indian friends “so that they understand…our values.” Some of this understanding lends itself to teasing and racial jokes that are still seen as acceptable considering they are coming from AIA peers. As one participant noted, “I like to be with Indian people because not in an insulting type of way but in a humorous way, I like to make Indian jokes with my friends. That's why I like having Indian friends. With American friends I could make some American jokes, but like I said, I like to be connected to India.” On the other hand, having Indian peers was stressful for some participants due to the sense of competition that inevitably arose. “Indians have this weird thing...where they're like two-sided. In front of me they're like...you're my best friend. Behind you, they just really want to compare themselves to you and get your opportunities and your first place” one participant stated. This example illustrates the pressures incurred by the internalization of the model minority myth, where Asian Americans must do well academically.

### Identity management

Identity seemed to be an area of particular struggle for these youth, as they straddled their “American” identity with their “Indian” identity. One participant captured this struggle effectively; “Well, if I go out in public in the world, then everyone will look at me as Indian. They will never look at me as Indian American. That's just how I look. So I think with physical appearance, I'm more Indian...Me personally, I'm Indian American because I know that. I know my experiences; they aren't Indian, they're Indian American.” This was also an issue in that some participants felt like they were deemed “too Indian” or “too American” in different contexts. For example, participants described Indians who “act American, or white” as being “white-washed” because they are “turning white” as being perceived as cool. “I've heard a few times, ‘oh you're Indian, but you're the cool kind…you're good Indian'.” For these participants, there is an implicit assumption that being Indian was not seen as desirable or socially acceptable.

Code-switching is a form of identity management observed among participants who reported actively “switching” identities based on the context and make-up of the peers with whom they interacted. For example, one participant stated “…the word Indian American, it means you live between two worlds, my experience. I come home, I'm Indian. I live Indian lives, I eat Indian food…I step over my threshold, I become American. Go to school, I'm an American...Your parents don't kind of understand the western world…and the western world doesn't really understand the Indian world….you live between two worlds and you've got to be knowledgeable to know how to balance them.” Similarly, another participant states that “on papers and everything, I write Asian American, but I don't know. With white people, I guess I'm American and then with Indian people I'm Indian.” One participant mentioned going to restaurants and making their “accent more American so that [non-Indians]...won't...think I can't speak English” when they order food. These participants shared experiences in juggling the two sets of values associated with each identity they managed.

Overall, participants seemed to have endorsed experiences of perceived discrimination of various levels and kinds even at young ages. Additionally, participants were able to verbalize their experiences related to developing their racial and ethnic identity. In particular, as second-generation AIA youth, it seems the discriminatory experiences and conflicting value systems made it challenging for these youth to maintain consistency in their identity development over time.

## Discussion

While the research is minimal, most of the available literature on the area of discrimination experiences among AIAs focuses on college-age and adult individuals. These studies have documented the negative impacts of discrimination on mental health outcomes, yet documentation of how this impacts adolescents is generally lacking (22, 28). Considering the historical context of AIAs immigrating over the last several decades, youth are likely to be experiencing stressors such as acculturation and navigating differing value systems. The purpose of the current study was to explore the unique discrimination experiences of AIA youth more broadly. Qualitative research designs have the benefit of providing a well-rounded understanding of a given area, which is what the current study aimed to capture for AIA youth's discrimination experiences.

This study was exploratory, allowing the participants to act as experts on their individual experiences of discrimination and identity formation. Participants provided information that was supportive of much of the research on discrimination experiences that South Asians (and in particular, AIAs) endure. The prompted scenarios in this study allowed for a “baseline” in understanding what each participant defined as discrimination in the first place. Interestingly, most participants seemed to have easily established an agreement in what was considered discriminatory. The younger participants struggled a little more with identifying microaggressive scenarios as discrimination, although they still acknowledged a level of discomfort with the situation.

The cross-case analysis pulled themes that further characterized the experiences these youth have with discrimination. In particular, participants were quick to share their affective responses to discrimination experiences. For example, these youth reported experiencing heightened sense of fear and anxiety about events they heard on the news and how they may feel at school with peers who actively created racially hostile environments.

It seems that AIA youth are constantly straddling the identity “line,” trying to balance their “Asian Indian” identity with their “American” identity. In the current study, participants discussed the various ways they found themselves “code-switching” to feel more comfortable with these opposing value systems. For these youth, this often meant having separate interests and behaviors at home with one's family vs. at school with friends. This implies that despite the “benefits” implied in assimilating into American culture, AIA adolescents may still be in a “lose-lose” situation where they are “too Indian” yet also “too American.” Additionally, most of the experiences these youth recounted were racially charged. This supports previous research that suggests second-generation youth endure more discrimination based on skin color and race, as opposed to the increased ethnic discrimination that their first-generation counterparts may experience (16).

Importantly, this study highlights a significant finding—that AIA youth are experiencing discrimination at a very early age. Although the participants in this study were adolescents, most participants shared that they were first aware of their race or ethnicity as early as preschool or elementary school, often due to a negative event that brought their race or ethnicity to the foreground. Second-generation AIA youth may be experiencing additional challenges considering the constant “tug-of-war” they experience with their Asian Indian and American identities. Further, the wide range of cultural, linguistic, historic, and religious diversity among AIAs as a whole group adds to the complexities of this identity development, and also leaves room for within-group discriminatory experiences.

## Limitations

While the current study has opened up avenues for better understanding the experiences of AIA youth, there are some limitations to consider when generalizing the findings. Participants were recruited through convenience sampling, and as such many of the participants were from a small sample in the Southwest region of the U.S. It is possible that the experiences outlined in their interviews are culturally bound to the Southwest; youth living in California among dense populations of SAAs may have completely different experiences with discrimination. Recruiting participants for interviews across a broader range of geographical areas in the United States may provide a more well-rounded picture of discrimination experiences.

## Conclusions

This study has provided an initial understanding of the experiences that may be shaping AIA youth's mental health and interactions with other AIAs or non-AIAs. Children and adolescents as young as 12 (and likely younger) are experiencing discrimination based on race and ethnicity. Further research is needed to determine how ethnic and racial identity may influence in how AIA youth relate to and navigate their worlds in the midst of ethnic and racial discrimination.

## Data availability statement

The datasets presented in this article are not readily available because data not to be made publicly available. Requests to access the datasets should be directed to drashaunni@gmail.com.

## Ethics statement

The studies involving human participants were reviewed and approved by Texas A&M University IRB. Written informed consent to participate in this study was provided by the participants' legal guardian/next of kin. Written informed consent was obtained from the minor(s)' legal guardian/next of kin for the publication of any potentially identifiable images or data included in this article.

## Author contributions

AU and JB contributed to study design. AU, JB, PS, and JL contributed to literature review and background. AU conducted data collection. AU and WL contributed to data analysis. All authors contributed to the article, expertise on the subject matter, and approving the submitted version.

## Funding

This study was supported by fellowships and dissertation grants from Texas A&M University to AU.

## Conflict of interest

The authors declare that the research was conducted in the absence of any commercial or financial relationships that could be construed as a potential conflict of interest.

## Publisher's note

All claims expressed in this article are solely those of the authors and do not necessarily represent those of their affiliated organizations, or those of the publisher, the editors and the reviewers. Any product that may be evaluated in this article, or claim that may be made by its manufacturer, is not guaranteed or endorsed by the publisher.
