# Supplementary material for: “No, but where are you really from?” Experiences of perceived discrimination and identity development among Asian Indian adolescents
Source: Front Public Health. 2022 Oct 18;10:955011. doi: 10.3389/fpubh.2022.955011 (PMC9623414; doi:10.3389/fpubh.2022.955011)
Supplement: Supplementary file 1 [file Data_Sheet_1.docx]

**Supplementary Material: Scenario Prompts**

1. Scenario #1: I’m going to tell you about some scenarios about things that happened between some random, fake people. They are not real. For the first scenario, a man, let’s say his name is Rajeev, is traveling to California for a business trip. Rajeev is a 26- year-old Indian businessman, and is wearing a button-down dress shirt with dress pants. He goes to the airport and is waiting in the security checkpoint line. He suddenly gets pulled aside for a random security “pat-down” although he is not carrying any questionable items. What do you think of Rajeev’s situation? What do you think is happening? How do you think he feels in this situation? Has anything similar to this ever happened to you or anyone you know? Please elaborate.
2. Scenario #2: For this second scenario, an older white male named Jonathan is speaking to his employee, Fatima, a young South Asian woman, about customer service and how to relate to potential customers. Jonathan warns Fatima that customers may be apprehensive about approaching her because they may feel intimidated. Fatima asked Jonathan why this may be, and Jonathan responds with, “because they probably feel like you are very…exotic. And that you could at any point sprout a few extra arms on the sides of your body!” What do you think of this situation? What do you think is happening? How do you think Fatima feels in this situation? Has anything like this ever happened to you or anyone you know? Please elaborate.
3. Scenario #3: For this third scenario, a young Indian man, Praveen, has just finished working out at the gym. He leaves the gym to go to his car, and begins reversing when a large Jeep filled with three or four young white males comes swerving around the corner, honking loudly. Praveen brakes his car abruptly, and looks outside of his window to take a look at the driver of the Jeep. The Jeep circles around and speeds by Praveen, and all four males proceed to show Praveen their middle fingers while simultaneously shouting “F**KING TERRORIST!” before they zoom off. What do you think of this situation? What do you think is going on? How do you think Praveen feels in this situation? Has anything like this ever happened to you or anyone you know? Please elaborate.
4. Scenario #4: In this scenario, Deepika, a 15-year-old Indian student, has just moved from New Jersey to Texas to start the 10th grade. She goes to the school to meet her counselor for the first time to create her schedule of classes. The counselor is a white woman in her 40’s, and she introduces herself slowly, enunciating all syllables and words carefully and loudly. When Deepika introduces herself, the counselor stands back in surprise and says “Wow! You speak English really well!” What do you think of this situation? What do you think is happening? How do you think Deepika feels in this situation? Has anything like this ever happened to you or anyone you know? Please elaborate.
5. Scenario #5**:** In this final scenario, a 17-year-old Indian woman named Yasmin goes to see a counseling therapist for treatment of anxiety. The therapist is a 50-year-old white woman who has had several years of experience working in the mental health field. The therapist begins asking Yasmin about her experiences and her background, and asks “You’re Hindu, right?” Yasmin corrected her and said “No, I’m Muslim.” What do you think of this situation? What do you think is happening? How do you think Yasmin feels in this situation? Has anything like this ever happened to you or anyone you know? Please elaborate.
6. Have you ever had any experiences where another person has said or done something (directly or indirectly) negative towards you based on your race or ethnicity? If so, please describe what happened? (Follow-up: was it verbal/non-verbal, a teacher, friend, stranger, family member?). How did you feel when that happened? What did you do/say in response? How did it impact your views about your own ethnic/racial group, if at all? Are there any other experiences you’d like to share?
